# Alteration of Intestinal Microbiota in Mice Orally Administered with Salmon Cartilage Proteoglycan, a Prophylactic Agent

**DOI:** 10.1371/journal.pone.0075008

**Published:** 2013-09-09

**Authors:** Krisana Asano, Sayuri Yoshimura, Akio Nakane

**Affiliations:** Department of Microbiology and Immunology, Hirosaki University Graduate School of Medicine, Hirosaki, Aomori, Japan; Charité-University Medicine Berlin, Germany

## Abstract

Proteoglycan (PG) extracted from salmon nasal cartilage has potential to be a prophylactic agent. Daily oral administration of the PG attenuates systemic inflammatory response in the experimental mouse models. In this study, we applied the culture-independent approach to investigate an alteration of intestinal microbiota composition in PG-administered mice. The results indicated that the population level of bacilli increased in the small and large intestine upon PG administration. On the other hand, the population level of clostridia decreased in the large intestine. The proportion of bacteria that are able to ferment saccharides and produce short-chain fatty acids increased in the small intestine and decreased in the large intestine. Importantly, population level of probiotic lactobacilli and bacteria exhibiting the immunomodulatory effect increased in the PG-administered mice. In addition, several disease-associated bacteria decreased upon PG administration. These results provided an understanding of the specific role of PG involved in host immune modulation and supported our hypothesis that daily oral administration of PG improves the overall balance in composition of the intestinal microbial community.

## Introduction

Proteoglycan (PG) consists of core protein with one or more covalently attached glycosaminoglycan chain(s). It is a component of extracellular matrix materials that exist in connecting tissues such as skin, bone, cartilage and vascular wall by forming a complex with collagen, fibronectin, laminin, hyaluronic acid and other glycoproteins. In corporation with collagen, fibronectin and laminin, PG has been shown to be involved in cellular proliferation and adhesion [1]. We have previously shown that PG extracted from salmon nasal cartilage has a potent effect on suppression of inflammatory responses induced by heat-killed *Escherichia coli* in mouse macrophages [2]. In addition, daily oral administration of PG attenuates the severity of experimental inflammatory colitis [3] and autoimmune encephalomyelitis [4]. Although our finding in both mouse models suggested that PG attenuates systemic inflammation by suppressing T-helper 17 (Th17) linage differentiation and inducing Foxp3^+^ regulatory T (Treg) cells, the mechanism of oral PG administration to regulate these T cells is still elusive.

Flora in the gastrointestinal tract (GIT) in mammals is highly complex and diverse. They have a profound effect on nutritional, physiological and immunological processes of the host [5]. Keeping these communities in balance is most likely crucial for health maintenance. Accumulating evidences suggested that an unfavorable alteration of the commensal structure of GIT microbiota implicates in chronic inflammatory bowel disease and also may be instrumental for the development of systemic immune diseases such as rheumatoid arthritis [6], autoimmune encephalomyelitis [7] and type-1 diabetes [8]. Therefore we hypothesized that the prophylactic effect of PG may result through the microbial communities in GIT. Between 40–60% of bacteria residing within the gut are reported to be un-culturable [9]. Thus in this study, the DNA-based culture-independent method was applied to analyze the composition of the GIT microbiota in PG-administered mice. Diversity and population levels of intestinal microbiota were compared between PG-administered and control mice. Bacterial phylotypes in the small and large intestine whose population levels were associated with PG-administration were investigated. The phylotypes that may have potential to regulate immune response upon daily PG administration were suggested.

## Materials and Methods

### Mice and ethics statement

C57BL/6 mice were purchased from Clea Japan, Inc., Tokyo, Japan and maintained under specific pathogen-free conditions at the Institute for Animal Experimentation, Hirosaki University Graduate School of Medicine. All experiments were carried out in strict accordance with the Guidelines for Animal Experimentation of Hirosaki University. The protocol was approved by the committee on the ethics of the Institute for Animal Experimentation, Hirosaki University Graduate School of Medicine (Permit number: M08028). For group A, mice were bred at the Institute for Animal Experimentation, Hirosaki University Graduate School of Medicine for 3 generations. For group B-group E, mice were purchased at different times and they were used after 1 week of purchase.

### PG administration and sample collection

PG extracted from salmon nasal cartilage was purchased from Kakuhiro Co., Ltd. (Aomori, Japan). It was dissolved in phosphate-buffered saline (PBS) at the concentration of 10 mg/ml. Six week-old female mice were administered with 200 µl of PG or PBS as control per os daily. To prevent fecal consumption between groups, mice administered with PBS and PG were housed in separate cages. On day 30, mice were starved for 16 h and then sacrificed by cervical dislocation. Luminal contents of small intestine (from 1 cm below stomach to 1 cm over caecum) and large intestine (from caecum to anus) were collected by gently scraping. Intestinal contents from 3 mice in each group were mechanically homogenized with sterile spatula and kept at -80^°^C until use.

### DNA isolation, 16S rRNA gene amplification and DNA sequencing

Frozen intestinal contents were thawed on ice and DNA was isolated using QIAamp DNA Stool Mini kit (QIAGEN, Hilden, Germany) according to the manufacturer’s instruction. Primer 27F (5’-AGAGTTTGATCMTGGCTCAG-3’, where M = C/A) and 519R (5’-GWATTACCGCGGCKGCTG-3’, where W = A/T and K = G/T) were used to amplify the variable region, V1 to V3, of 16S rRNA gene [10]. Primer 27F and 519R were tagged with a multiplex identifier (MID) sequence (Table S1) at 5’ end along with adapter sequence (5’-CGTATCGCCTCCCTCGCGCCATCAG-3’ and 5’-CTATGCGCCTTGCCAGCCCGCTCAG-3’, respectively) to allow all samples to be included in a single FLX Titanium sequencing plate. PCR reaction was performed using FastStart High Fidelity PCR system (Roche Diagnostics, Indianapolis, IN) and PCR Nucleotide Mix (Roche Diagnostics). The conditions were 95°C for 2 min, 35 cycles of 95°C for 30 sec, 55°C for 30 sec and 72°C for 30 sec, followed by 72°C for 4 min. DNA products were quantified and pooled for sequencing with the Genome Sequencer FLX Titanium system (Roche Diagnostics) at InfoBio Co., Ltd., Tokyo Japan (http://www.infobio.co.jp).

### Sequence analysis

The 16S rRNA gene sequences reported herein have been deposited in the DDBJ Sequence Read Archive (accession number of submission; DRA000939 and DRA001079, accession number of study; DRP000976 and DRP001126, accession numbers of samples; DRS005602 to DRS005609 and DRS011995 to DRS012006, accession numbers of experiments; DRX005456 to DRX005463 and DRX012124 to DRX012135; accession numbers of runs; DRR006232 to DRR006239 and DRR013297 to DRR013308). The sequences from each labeled-tag were clustered using a sequence similarity at 96.00% with the software of cdhit-v4.5.4. The clusters less than 350 nucleotides in length were discarded. The representative sequence of each cluster was searched for homology using BLASTN against Genbank database extracted as a product name of 16S rRNA or 16S ribosomal RNA with the length of more than 299 bases. The taxon for each representative sequence was assigned from the closest related 16S rRNA sequence with a minimum alignment length of 350 nucleotides and minimum percent identity of 85.0. Percent relative count of each taxon was calculated from count number of each specific taxon and total count number in each sample.

### Statistical analysis

Associations between bacterial phylotypes and PG administration were examined by Fisher exact test. *P* values less than 0.05 were used to indicate the statistical difference of bacterial counts between PG-administered and control mice.

## Results

### Numbers of 16S rRNA gene sequence and taxonomic assignment

**Figure 1:**
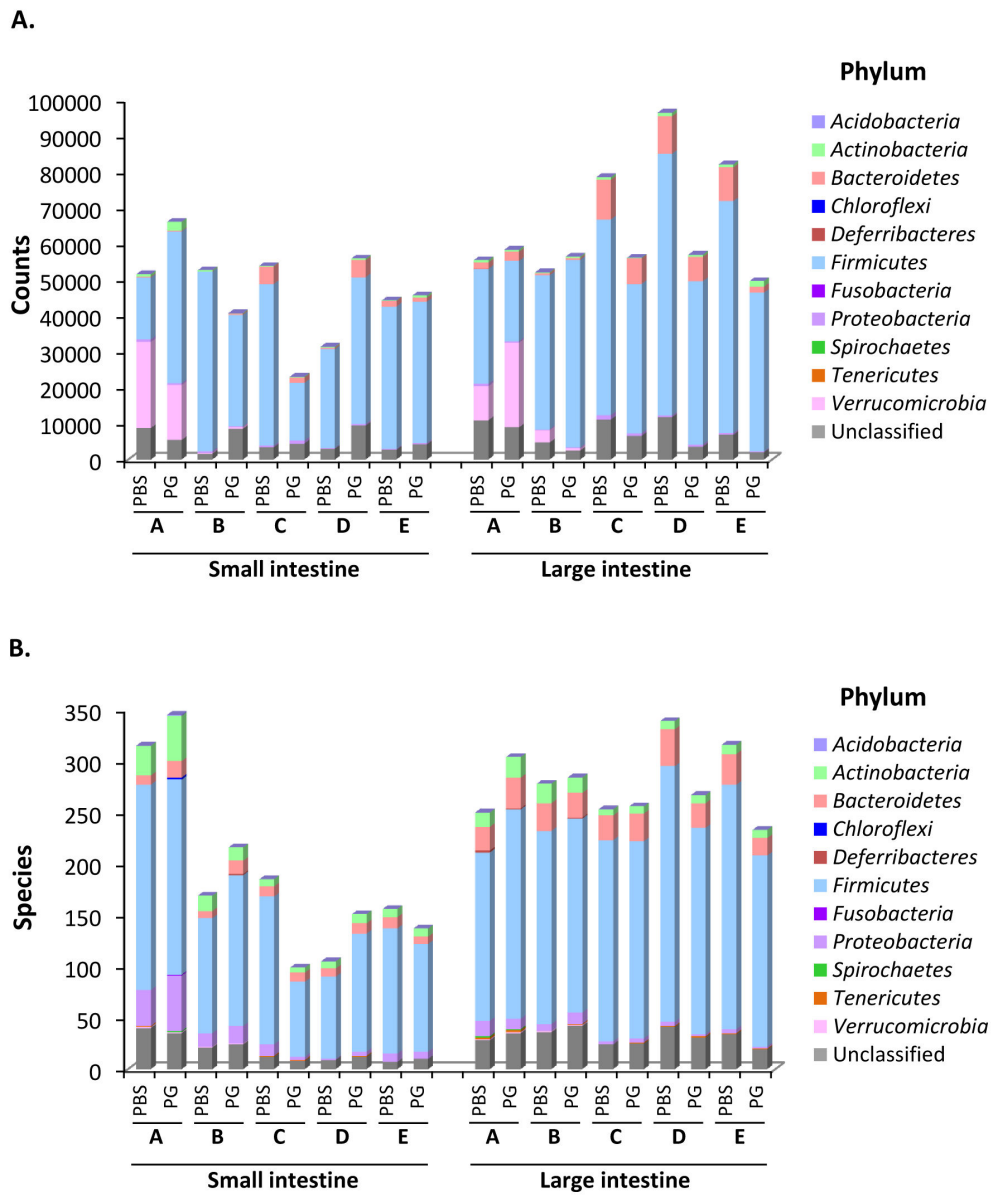
Total counts (A) and number of assigned phylotypes (B) obtained from each intestinal sample. (A) Total counts were obtained after removing the sequences under threshold of 350 nucleotides in length and 85.0% identity. (B) Bacterial phylotypes from each sample were assigned from the closest related 16S rRNA sequence. The number of counts and the number of bacterial phylotypes in each phylum were indicated. Sequences that could not be classified into known phyla were assigned as ‘Unclassified’. A, B, C, D and E indicate the results obtained from group A, group B, group C, group D and group E mice, respectively.

**Figure 2:**
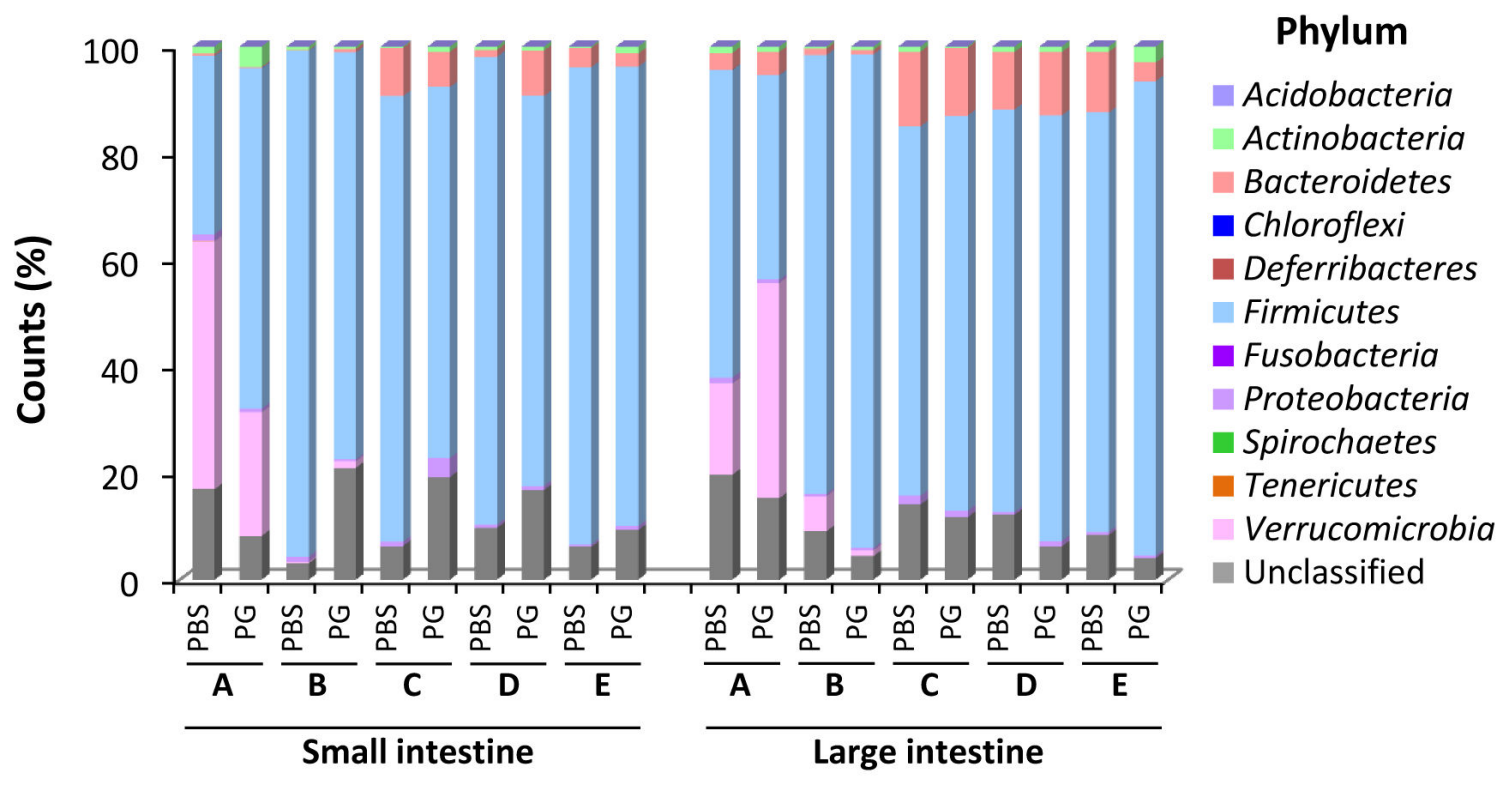
Relative abundance at phylum-level of GIT microbiota in PBS- and PG-administered mice. Relative count of each phylum was calculated from counts of specific phylum and total counts of each sample. Sequences that could not be classified into any known bacterial phyla were assigned as ‘Unclassified’. A, B, C, D and E indicate the results obtained from group A, group B, group C, group D and group E mice, respectively.

From DNA sequencing data generated by the FLX Titanium Sequencer, a total of 1,171,101 counts were obtained from 20 samples. As shown in Figure S1, the sequences longer than 349 nucleotides in length were predominant in group A and B. Thus the sequences having less than 350 nucleotides were discarded. The bacterial phylotype or taxon for the representative sequence was then assigned from the closest related 16S rRNA sequence with threshold of 350 nucleotides in alignment length and 85.0% identity. Total count numbers with or without threshold from each sample are listed in Table S1. A total of 1,107,491 sequences with acceptable threshold were obtained from 20 samples with an average of 55,375 counts (range: 23,042-96,496) per sample (Table S1 and Figure 1A). The data indicated that 94.6% of total sequences could be assigned to domain Bacteria. The variety of phylotypes assigned from each sample is shown in Figure 1B. The relative alteration of the bacterial variety upon PG administration was not found among all 5 groups. The results suggested that there was not an association between PG administration and biodiversity of GIT microbiota. The distribution of intestinal bacteria at phylum-level is shown in Figure 2 and Table S2-Table S3. Excluding unclassified phylum, majority of GIT microbial population belonged to 5 phyla, *Actinobacteria*, *Bacteroidetes*, *Firmicutes*, *Proteobacteria* and *Verrucomicrobia*. However, the member in phylum *Verrucomicrobia* was found in only group A and group B. To address the effect of salmon cartilage PG on intestinal microbiota, bacterial phylotypes whose population level showed relative alteration at least in 4 of 5 groups were investigated. As shown in Figure 2 and Table S2-Table S3, the population of phylum *Actinobacteria* and unclassified phylum in the small intestine and the population of phylum *Firmicutes* in the large intestine showed relative increasing upon PG administration. On the other hand, the population of phylum *Firmicutes* in the small intestine and the population of unclassified phylum in the large intestine showed negative association with PG. We further analyzed the alterations in the population levels of each taxon. Distribution of all intestinal phylotypes in each phylum is given in Materials S1. Obviously, the population of class *Bacilli* increased in both small and large intestine. On the other hand, the population of class *Clostridia* decreased in the large intestine (Table S4-Table S5).

### In the small intestine, the population level of saccharolytic bacteria, *Lactobacillus* *intestinalis* and bacteria exhibiting immunomodulatory effect increased upon oral administration of PG.

**Figure 3:**
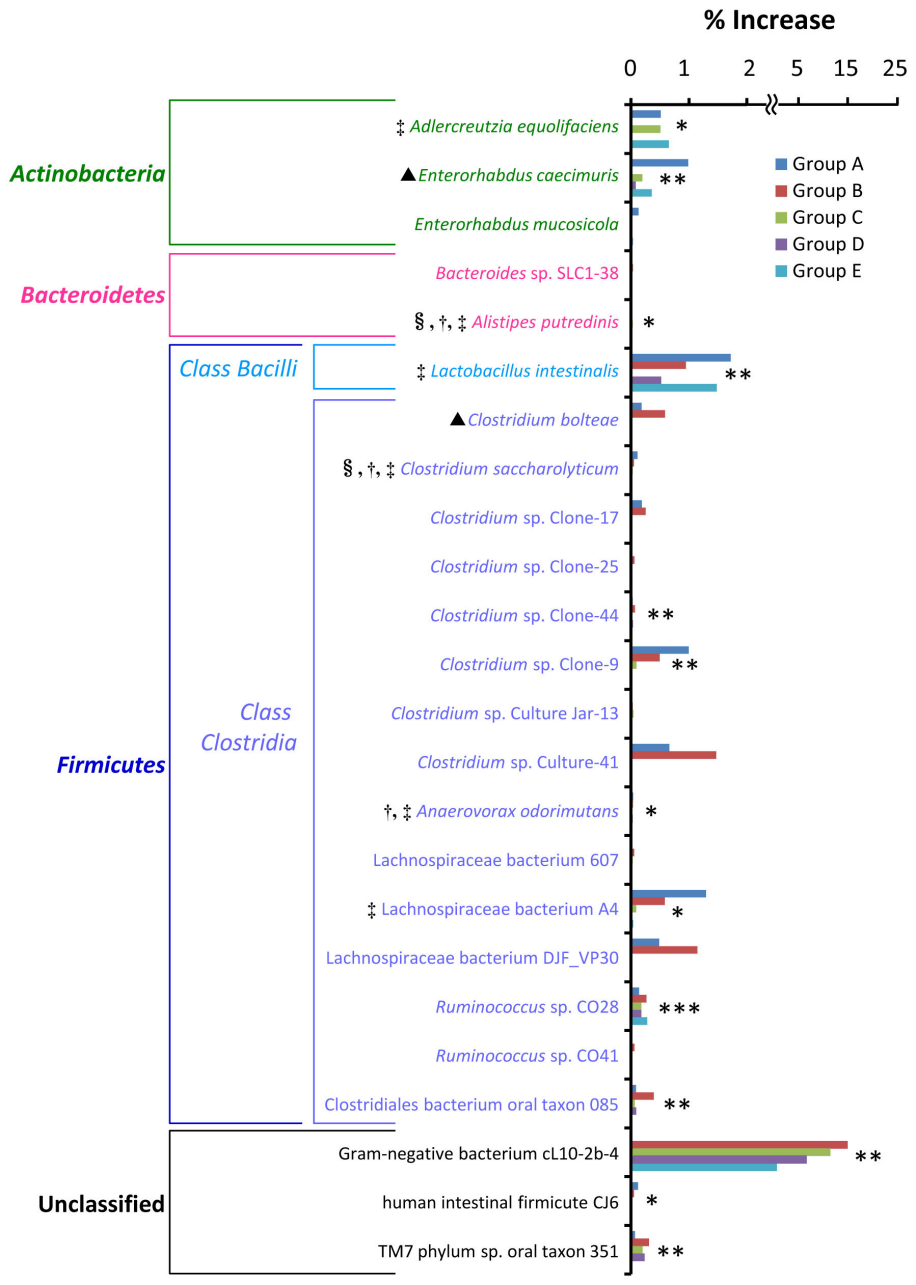
Bacterial phylotypes in the small intestine whose population level increased upon PG-administration. Percent increase was calculated by subtracting the percent population level of each specific phylotype found in the PG-administered mice with that found in the PBS control mice. Associations between bacterial phylotypes and PG administration were examined by Fisher exact test. The phylotypes having *P* values less than 0.05 at least in 2 groups (see Table S6) are shown. Asterisks *, ** and *** indicate bacterial phylotypes having *P* values less than 0.05 in 3, 4 and 5 groups, respectively. §: Saccharolytic bacteria, †: short-chain fatty acids-producing bacteria, ‡: bacteria with immunomodulatory effect, ▲: disease-associated bacteria.

There were 30 phylotypes whose population level increased in the small intestine upon PG administration, relating at least in 4 groups of mice (Table S6). After statistical analysis, there were 24 phylotypes showing a statistical difference in population between PG-administered and control mice (*P* values less than 0.05) at least in 2 groups (Table S6 and Figure 3). Among these populations, majority of phylotypes have not yet been characterized and classified, although they are found as normal flora in humans or animals. They were *Bacteroides* sp. SLC1-38, *Clostridium* sp. Clone-17, *Clostridium* sp. Clone-25, *Clostridium* sp. Clone-44, *Clostridium* sp. Clone-9, *Clostridium* sp. Culture Jar-13, *Clostridium* sp. Culture-41, Lachnospiraceae bacterium 607, Lachnospiraceae bacterium DJF_VP30, *Ruminococcus* sp. CO28, *Ruminococcus* sp. CO41, Clostridiales bacterium oral taxon 085, Gram-negative bacterium cL10-2b-4, human intestinal firmicute CJ6 and TM7 phylum sp. oral taxon 351. Obviously, several phylotypes whose population level increased upon PG administration have been shown to provide a health benefits on the hosts. *Alistipes putredinis* [11] and *Clostridium* *saccharolyticum* [12] are able to utilize carbohydrates or polysaccharides as carbon source. These bacteria are able to produce acetate, propionate and butyrate as fermented products. *Adlercreutzia* *equolifaciens* has ability to produce equol [13], a nonsteroidal estrogen which has beneficial effects on the incidence of prostate cancer [14] and reduces experimental cutaneous inflammation in mice [15]. *Lactobacillus* *intestinalis* in class *Bacilli*, phylum *Firmicutes* is a potential probiotic strain [16]. *Anaerovorax* *odorimutans* is a putrescine-fermenting bacterium which changes putrescine to acetate, butyrate, and ammonia as metabolic products [17]. Lachnospiraceae bacterium A4 is negatively associated with development of colitis [18]. Excluding the phylotypes mentioned above, *Enterorhabduscaecimuris* has been isolated from mice with spontaneous colitis [19]. *Clostridium bolteae* has been shown to be overabundant in the intestinal tract of autistic children suffering from gastric intestinal ailments [20]. They probably associate with the disease.

### Population level of only 4 phylotypes including a disease-associated bacterium decreased in the small intestine upon PG administration.

**Figure 4:**
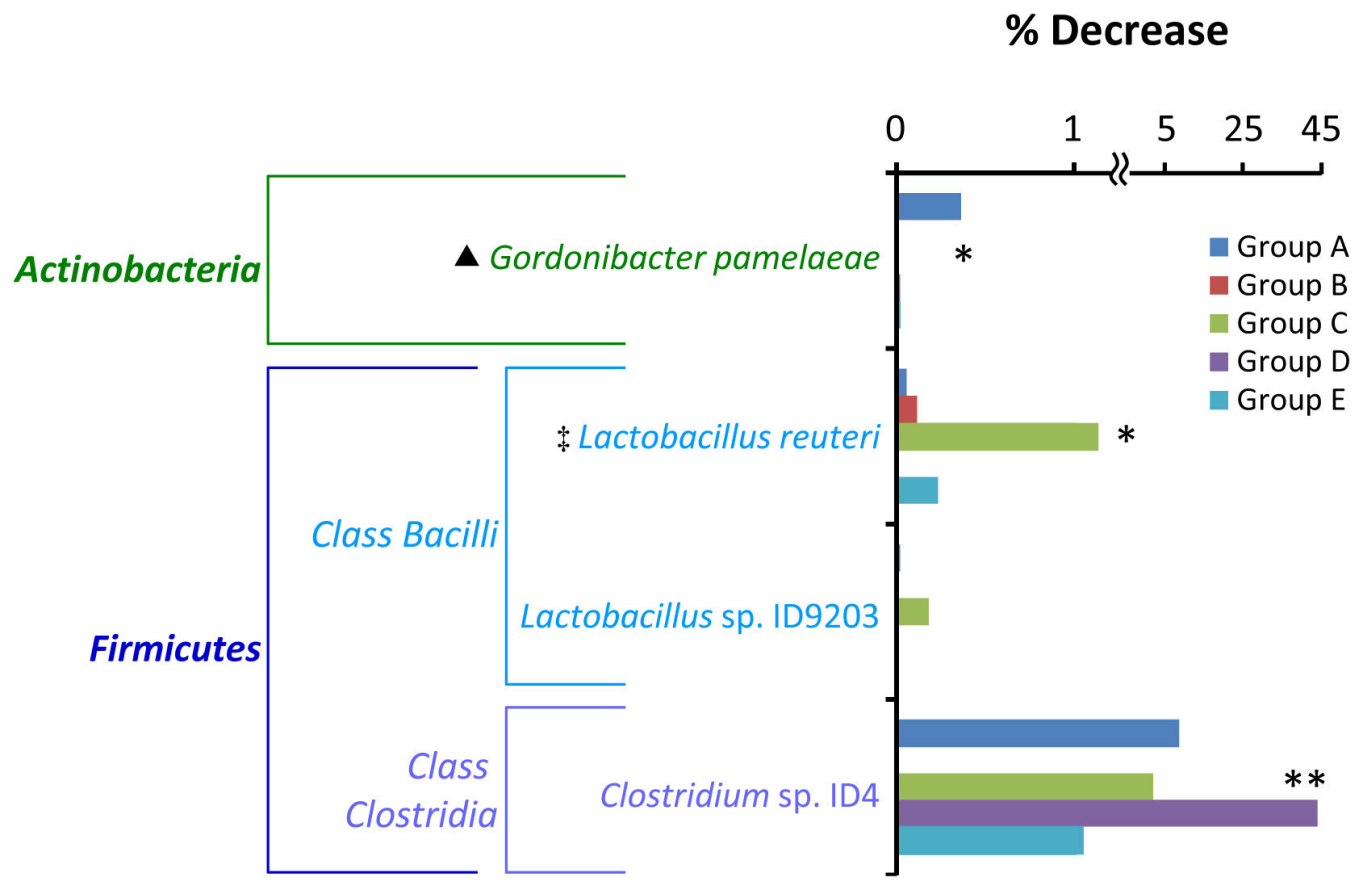
Bacterial phylotypes in the small intestine whose population level decreased upon PG-administration. Percent decrease was calculated by subtracting the percent population level of each specific phylotype found in the PBS control mice with that found in the PG-administered mice. Associations between bacterial phylotypes and PG administration were examined by Fisher exact test. The phylotypes having *P* values less than 0.05 at least in 2 groups (see Table S7) are shown. Asterisks * and ** indicate bacterial phylotypes having *P* values less than 0.05 in 3 and 4 groups, respectively. ‡: Bacteria with immunomodulatory effect, ▲: disease-associated bacteria.

At least in 4 groups of mice, there were 6 phylotypes whose population level relatively decreased in the small intestine upon PG administration (Table S7) and only 4 phylotypes showing a statistical difference in population between PG-administered and control mice (*P* values less than 0.05) at least in 2 groups (Figure 4). Among these populations, *Clostridium* sp. ID4 is only one phylotype belonging to class *Clostridia* phylum *Firmicutes*. The biological properties and species of this strain have not yet been characterized. Two phylotypes in this population belong to genus *Lactobacillus*. They are probiotic *Lactobacillus reuteri* [21] and folate-producing *Lactobacillus* sp. ID9203 (referred to 16S rRNA gene GenBank: AY862434). *Gordonibacterpamelaeae* in phylum *Actinobacteria* has been shown to associate with diseases. It has been isolated from patients with Crohn’s disease and rectosigmoid carcinoma [22,23].

### Prevalence proportion of probiotic lactobacilli and bacteria with immunomodulatory effect was enhanced in the large intestine upon PG administration.

**Figure 5:**
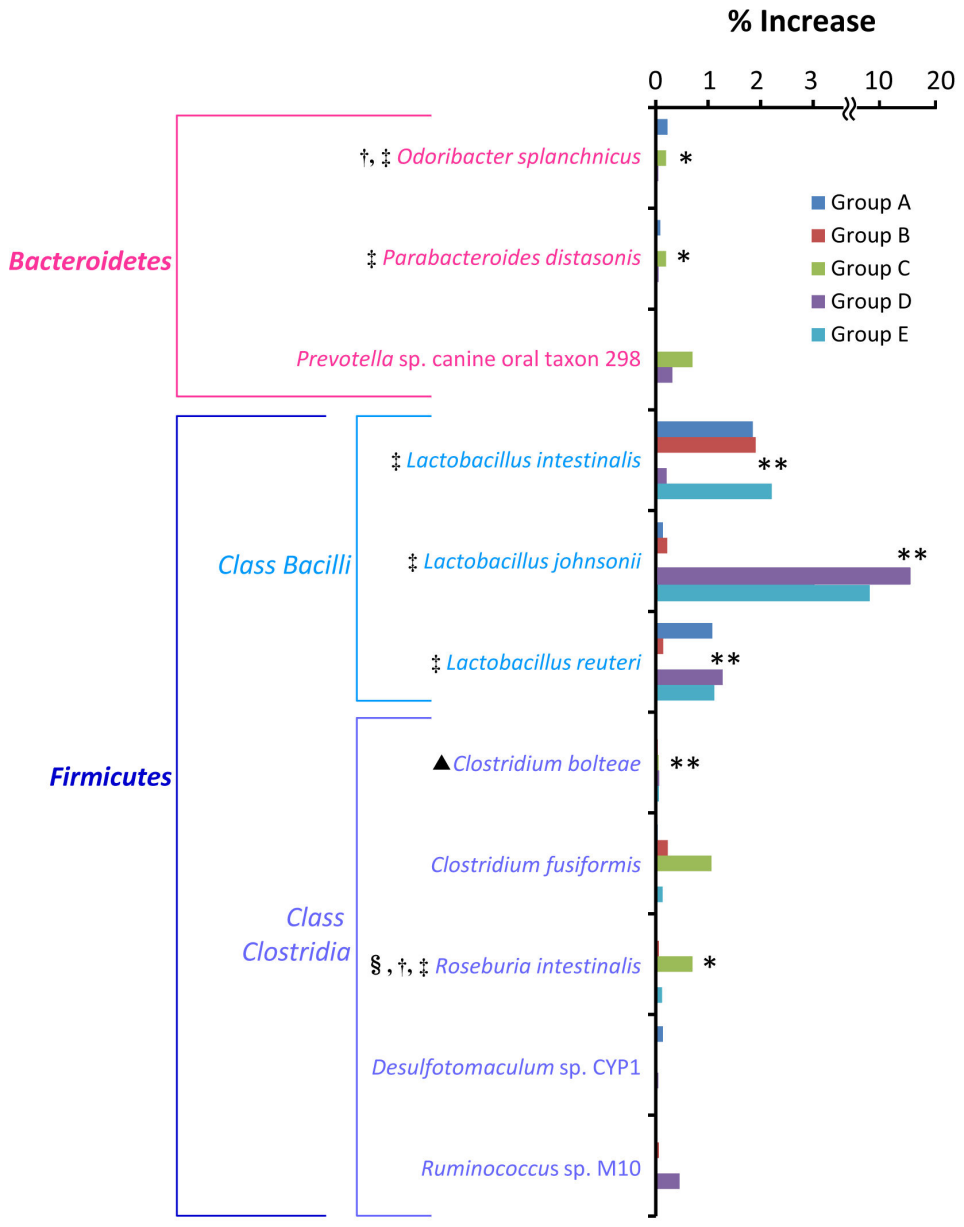
Bacterial phylotypes in the large intestine whose population level increased upon PG-administration. Percent increase was calculated by subtracting the percent population level of each specific phylotype found in the PG-administered mice with that found in the PBS control mice. Associations between bacterial phylotypes and PG administration were examined by Fisher exact test. The phylotypes having *P* values less than 0.05 at least in 2 groups (see Table S8) are shown. Asterisks * and ** indicate bacterial phylotypes having *P* values less than 0.05 in 3 and 4 groups, respectively. §: Saccharolytic bacteria, †: short-chain fatty acids-producing bacteria, ‡: bacteria with immunomodulatory effect, ▲: disease-associated bacteria.

At least in 4 groups of mice, there were 16 phylotypes whose population level relatively increased in the large intestine upon PG administration (Table S8). Only 11 phylotypes had a statistical difference in population between PG-administered and control mice (*P* values less than 0.05) at least in 2 groups (Table S8 and Figure 5). The role of *Prevotella* sp. canine oral taxon 298, *Desulfotomaculum* sp. CYP1 and *Ruminococcus* sp. M10 in host immune response is unknown. *Odoribacter* *splanchnicus* is a butyric acid-producing bacterium which is reclassified from *Bacteroides* *splanchnicus* [24,25]. *Parabacteroides* *distasonis* is a bacterium which has been shown to attenuate experimental murine colitis through modulation of immunity [26]. *Lactobacillus* *intestinalis* [16], *Lactobacillus* *johnsonii* [27] and *Lactobacillus reuteri* [21] are a characterized by various probiotic properties. *Clostridium* *fusiformis* is a strain of gut barrier flora against pathogen, *Clostridium difficile* in mouse model (referred to 16S rRNA gene GenBank: AF028349). *Roseburia* *intestinalis* is saccharolytic, butyrate producing bacterium [28]. It corresponds to a reduction of plasma interleukin-6 [29].

Besides these bacteria, only one phylotype probably associates with disease that is *Clostridium bolteae*. As mentioned above, overabundance of this bacterium is found in the intestinal tract of autistic children suffering from gastric intestinal ailments [20].

### Population level of saccharolytic clostridia and disease-associated bacteria decreased in the large intestine of PG-administered mice.

**Figure 6:**
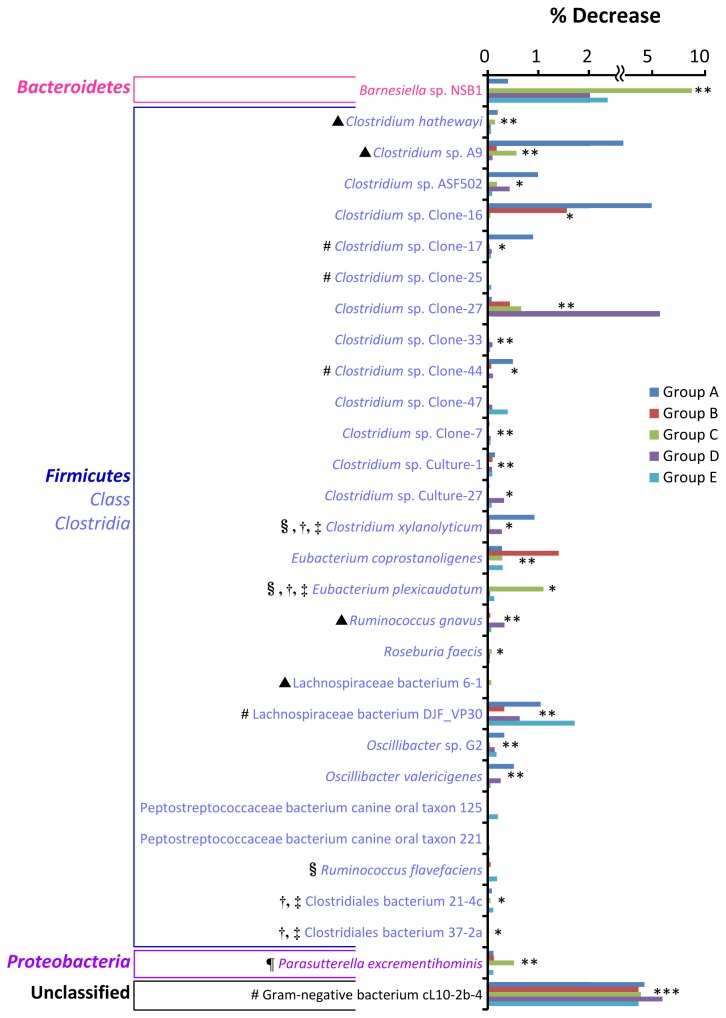
Bacterial phylotypes in the large intestine whose population level decreased upon PG-administration. Percent decrease was calculated by subtracting the percent population level of each specific phylotype found in the PBS control mice with that found in the PG-administered mice. Associations between bacterial phylotypes and PG administration were examined by Fisher exact test. The phylotypes having *P* values less than 0.05 at least in 2 groups (see Table S9) are shown. Asterisks *, ** and *** indicate bacterial phylotypes having *P* values less than 0.05 in 3, 4 and 5 groups, respectively. #: Bacteria whose population level also increased in the small intestine (see Figure 3), §: saccharolytic bacteria, †: short-chain fatty acids-producing bacteria, ‡: bacteria with immunomodulatory effect, ¶: asaccharolytic bacteria, ▲: disease-associated bacteria.

At least in 4 groups of mice, there were 42 phylotypes whose population level decreased in the large intestine upon PG administration (Table S9). Only 30 phylotypes had a statistical difference in population between PG-administered and control mice (*P* values less than 0.05) at least in 2 groups (Table S9 and Figure 6). Among these populations, major phylotypes (27 phylotypes) are members in class *Clostridia* phylum *Firmicutes*. The role in GIT immunity of the predominant phylotypes including *Barnesiella* sp. NSB1, *Clostridium* sp. Clone-strains, *Clostridium* sp. Culture-strains, *Eubacterium* *coprostanoligenes* , Lachnospiraceae bacterium DJF_VP30, *Oscillibacter* species, Peptostreptococcaceae bacterium canine oral taxon 125 and 221, and Gram-negative bacterium cL10-2b-4 is unclear. Obviously, the proportion of 5 phylotypes also increased in the small intestine upon PG administration. They are *Clostridium* sp. Clone-17, *Clostridium* sp. Clone-25, *Clostridium* sp. Clone-44, Lachnospiraceae bacterium DJF_VP30 and Gram-negative bacterium cL10-2b-4 (Figure 3).

Among these 30 phylotypes, one phylotype belongs to phylum *Proteobacteria* that is *Parasutterella* *excrementihominis* . *Parasutterella* *excrementihominis* is a member in class *Betaproteobacteria*. It is a strictly anaerobic, non-spore-forming, Gram-negative coccobacillus found in human feces. Biochemically, this strain is largely unreactive as well as asaccharolytic [30]. On the other hand, 5 strains whose population level decreased upon PG administration are able to digest saccharides and/or produce butyrate. They are *Clostridium* *xylanolyticum* [31], *Eubacterium* *plexicaudatum* [32], *Ruminococcus* *flavefaciens* [33], Clostridiales bacterium 21-4c [34] and Clostridiales bacterium 37-2a [34].

Interestingly, 4 phylotypes whose population level decreased in the large intestine have been shown to be associated with diseases. *Clostridium hathewayi* causes bacteremia and septicemia [35,36]. Multiple studies have identified that *Ruminococcus* *gnavus* is enriched-bowel inflammatory disease [37]. *Clostridium* sp. A9 and Lachnospiraceae bacterium 6-1 are flagellated bacteria which implicate in Crohn’s disease (referred to 16S rRNA gene GenBank: DQ789119.1, DQ789124 and DQ789123, respectively)*.*

## Discussion

Intestinal microbiota constitutively and profoundly affects the development and balance of the host immune system. They have been implicated in prevention of damage induced by opportunistic microbes, in repair of damage to mucosal barrier and in influencing systemic autoimmune diseases. The composition of a host’s intestinal microbiota directs the type of mucosal and systemic immune response by affecting the proportion and number of functionally distinct T cells subsets. In particular, the microbiota composition affects the differentiation of intestinal Th17 cells and Treg cells, both of which play crucial roles in maintaining mucosal barrier of functions and in controlling immunological homeostasis [38]. We have previously reported that salmon cartilage PG has a potential to be a prophylactic agent because it exhibited immunomodulatory activity by suppressing systemic host inflammation in colitis and autoimmune encephalomyelitis [3,4]. In both models, Th17 cells are important for progression of the diseases [39,40]. From the chemokine and cytokine production as well as T cell analysis, we found that salmon cartilage PG suppresses Th17 linage differentiation and enhances of Treg expansion [3,4]. To find the link between oral administration of salmon PG and regulation of these T cells, the composition of GIT microbiota in PG-administered mice was investigated in this study.

The bar-coded pyrosequencing technique is culture-independent molecular tool that is widely used to analyze the composition of the GIT microbiota. It has been proven that this technique can be applied for quantitative analysis. In an artificial sample, number of reads correlated approximately with the number of encoded 16S rRNA gene and viable counts of bacteria [41]. In order to avoid the artificial environmental factors which may have influence on the composition of intestinal microbiota, 5-indendent groups of mice from different environments (group A-group E) were used in our experiments. Although only 5 independent experiments were performed, each intestinal sample was pooled from 3 mice. More than 1,000,000 counts were obtained from a total of 30 mice in all 5 experiments (Figure 1A and Table S1). These numerous data were reliable to suggest the phylotypes that have potential to regulate host immune response upon daily PG administration.

As shown in Figure 1A and Table S1, between 23,042 and 96,496 counts were obtained from each sample and more than 94% of total counts could be assigned to domain Bacteria. These results suggested that our methods for sample preparation, 16S rRNA gene amplification, and sequencing were effective. Distribution of bacterial population in each phylum (Figure 2) demonstrated that compositions of GIT microbiota between each group are basically different. These data correlated with previous study which has shown that environment has an influence on variation of gut microbiota [42].

Due to the variation of gut microbiota in each group of mice, many phylotypes were not found among all samples (Materials S1). Thus the alteration of gut microbiota upon PG administration correlated among all 5 groups was almost absent (data not shown). To address the effect of salmon cartilage PG on intestinal microbiota communities, we investigated the bacterial phylotypes showing relative alterations at least in 4 groups. Associations between bacterial phylotypes and PG administration were then examined by Fisher exact test (Table S6-Table S9). The phylotypes with significant difference (*P* values less than 0.05) at least in 2 groups were considered as potent associated strains upon PG administration (Figure 3-Figure 6).

The majority of bacteria whose population was altered upon PG administration belong to phylum *Firmicutes*. The population level of class *Bacilli* increased in both small and large intestine and the population level of class *Clostridia* decreased in the large intestine (Table S4-Table S5). Excluding non-characterized bacteria, major populations which increased in the small intestine and decreased in the large intestine were saccharolytic bacteria (Figure 3 and Figure 6). These bacteria are able to use saccharides and/or starch as fermentable substrates. They primarily produced short-chain fatty acids such as acetate, propionate and butyrate by anaerobic bacterial fermentation of undigested dietary carbohydrates and polysaccharides. PG contains glycosaminoglycan chains in its structure, thus it is expected to be a fermentable substrate and promote growth of these bacteria. It has been shown that oligosaccharides are preferentially fermented in upper part of the large intestine [43]. This may be a reason that the effect of PG on growth of these bacteria was found only in small intestine but not in the large intestine. Short-chain fatty acids-producing bacteria perform a number of activities important in supporting the normal function of GIT tract [44–46]. Acetate and propionate have been shown to be effective anti-inflammatory and immune-modulating agents in human colon cancer cell lines and mouse models [47]. Especially, butyrate is an important energy source for colonic epithelial cells. It has been shown to enhance epithelial barrier integrity and modulate the gastrointestinal immune response [48]. Furthermore, butyrate provides a defense against cancer and ulcerative colitis in humans and animals [49].

Growth and metabolisms of saccharolytic producing bacteria may then have an influence on the population level of other bacteria. The proportion of bacteria that provide health benefits on hosts increased in the small and large intestine upon PG administration. Lactobacilli in class *Bacilli* phylum *Firmicutes* have been reported to have probiotic properties [16,21,27]. Treatment of mice with *Lactobacillus reuteri* has been shown to increase the percentage of Treg cells [21]. Many bacteria whose population increased in the small and large intestine exhibit immunomodulatory effect. They included equal-producing *Adlercreutzia* *equolifaciens* [13–15], Lachnospiraceae bacterium A4 [18], *Parabacteroides* *distasonis* [26], *Clostridium* *fusiformis* and *Roseburia* *intestinalis* [28,29]. In contrast, the proportion of disease-associated phylotypes decreased in the small and large intestine upon PG administration. They included *Gordonibacterpamelaeae* [22,23], *Clostridium hathewayi* [35,36], *Ruminococcus* *gnavus* [37], *Clostridium* sp. A9 and Lachnospiraceae bacterium 6-1.

It should be noted that the population of some disease-associated bacteria increased (Figure 3 and Figure 5) and the population of some bacteria with immunomodulatory effect decreased (Figure 4 and Figure 6) upon PG administration. The reason for these changes in view of the immune response is unclear. However, host immunological homeostasis is controlled by the balance between of Th17 and Treg cells [38]. Thus, this bacterial proportion may play a role in maintaining this balance. In addition, many GIT bacteria whose population levels altered upon PG administration were uncharacterized and unidentified to species level (Figure 3-Figure 6). Although the role of these bacteria in host immunity is unknown, these bacteria may also play an important role in balancing of GIT bacterial community.

From our results, we conclude that daily oral PG administration induces the compositional alteration of GIT microbiota. Due to utilization of PG as nutritional source, the proportion of saccharide-degrading and short-chain fatty acids-producing bacteria increased especially in the small intestine. Moreover, daily oral PG administration reduced the proportion of several bacteria that are associated with diseases. Concurrently, PG administration also enhanced the proportion of probiotic lactobacilli and bacteria with immunomodulatory effect. In the present study, our results suggested that the effect of salmon cartilage PG on homeostasis of host immunity is mediated through the overall balance in the composition of gut microbial community. This study provided the understanding of the specific role of PG involved in host immune modulation.

## Supporting Information

Figure S1 **Counts of DNA sequences obtained from group A and B mice with various nucleotides in length generated by the FLX Titanium Sequencer.** The sequences longer than 349 nucleotides in length were predominant.
(TIF)

Table S1 **MID sequence, number of total counts and number of phylotypes obtained from each sample.**
(DOCX)

Table S2 **Distribution of the intestinal bacteria in the small intestine of PBS- and PG-administered mice at phylum-level.**
(DOCX)

Table S3 **Distribution of the intestinal bacteria in the large intestine of PBS- and PG-administered mice at phylum-level.**
(DOCX)

Table S4 **Distribution of mouse intestinal bacteria belonging to phylum *Firmicutes* in the small intestine of PBS- and PG-administered mice.**
(DOCX)

Table S5 **Distribution of mouse intestinal bacteria belonging to phylum *Firmicutes* in the large intestine of PBS- and PG-administered mice.**
(DOCX)

Table S6 **Bacterial phylotypes whose population level increased upon PG administration in the small intestine.**
(DOCX)

Table S7 **Bacterial phylotypes whose population level decreased upon PG administration in the small intestine.**
(DOCX)

Table S8 **Bacterial phylotypes whose population level increased upon PG administration in the large intestine.**
(DOCX)

Table S9 **Bacterial phylotypes whose population level decreased upon PG administration in the large intestine.**
(DOCX)

Materials S1 **Distribution of all intestinal phylotypes in domain, phylum, class, order, family, genus and species.**
(XLSX)
