# Supplementary figures and images for: Alteration of Intestinal Microbiota in Mice Orally Administered with Salmon Cartilage Proteoglycan, a Prophylactic Agent
Source: PLoS One. 2013 Sep 9;8(9):e75008. doi: 10.1371/journal.pone.0075008 (PMC3767651; doi:10.1371/journal.pone.0075008)

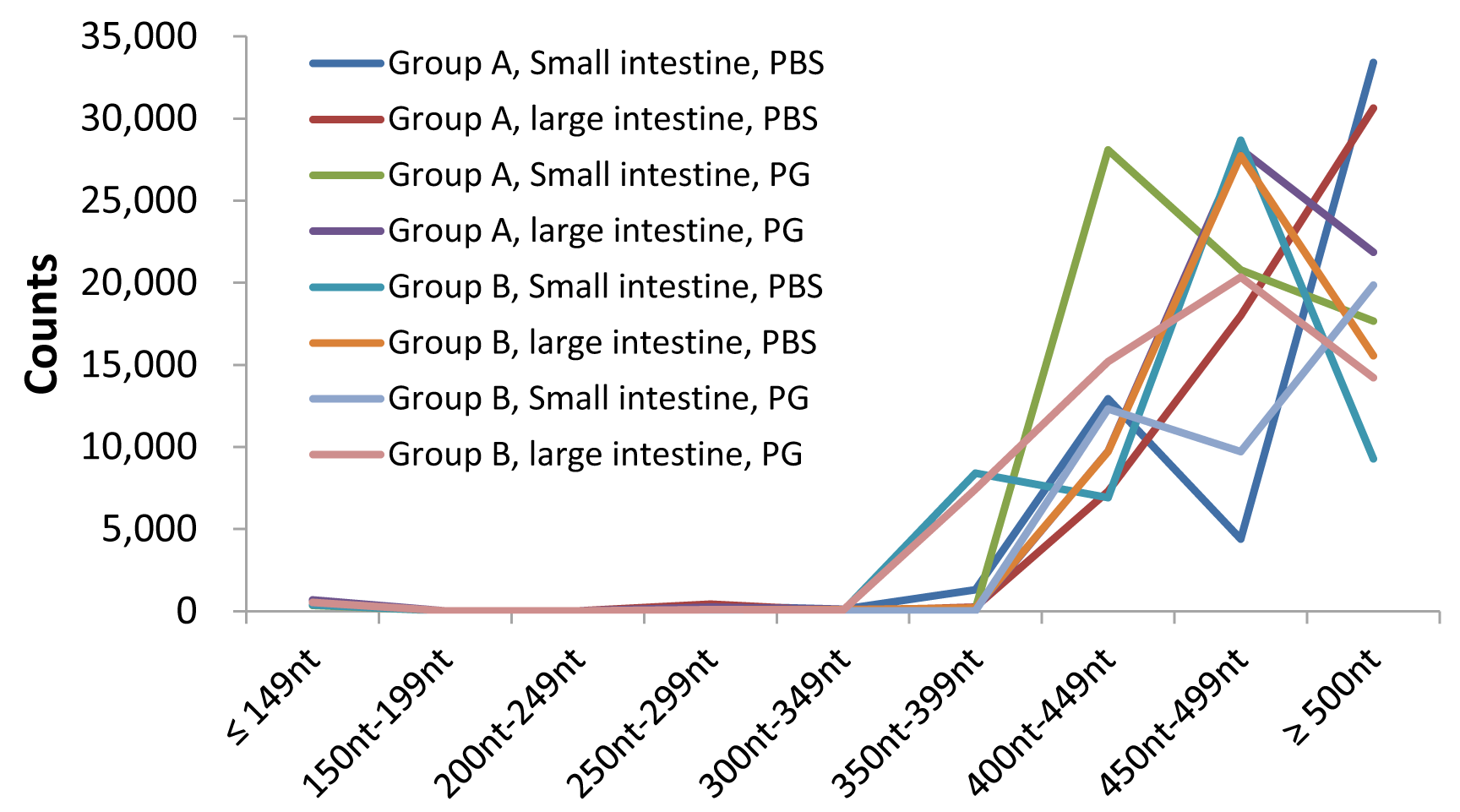

Supplement: Figure S1 — Counts of DNA sequences obtained from group A and B mice with various nucleotides in length generated by the FLX Titanium Sequencer. The sequences longer than 349 nucleotides in length were predominant. (TIF) [file pone.0075008.s001.tif]
