# Supplementary material for: Alteration of Intestinal Microbiota in Mice Orally Administered with Salmon Cartilage Proteoglycan, a Prophylactic Agent
Source: PLoS One. 2013 Sep 9;8(9):e75008. doi: 10.1371/journal.pone.0075008 (PMC3767651; doi:10.1371/journal.pone.0075008)
Supplement: Table S1 — MID sequence, number of total counts and number of phylotypes obtained from each sample. (DOCX) [file pone.0075008.s002.docx]

Table S1. MID sequence, number of total counts and number of phylotypes obtained from each sample.

| **Sample** | | | **MID sequence^a^** | **Number of total counts without threshold** | **Number of total counts with threshold** | **% of total counts with threshold** | **Number of phylotypes** |
| --- | --- | --- | --- | --- | --- | --- | --- |
| **Group** | **Administration** | **Intestine** |  |  |  |  |  |
| **A** | PBS | Small | ACGAGTGCGT | 52,918 | 51,569 | 97.5 | 315 |
|  |  | Large | CGTGTCTCTA | 57,249 | 55,480 | 96.9 | 250 |
|  | PG | Small | TACACGTGAT | 67,317 | 66,123 | 98.2 | 345 |
|  |  | Large | TGTACTACTC | 60,977 | 58,392 | 95.8 | 304 |
| **B** | PBS | Small | TACACACACT | 53,700 | 52,654 | 98.1 | 169 |
|  |  | Large | ACGCTCGACA | 53,946 | 52,088 | 96.6 | 278 |
|  | PG | Small | CTCGCGTGTC | 42,453 | 40,721 | 95.9 | 216 |
|  |  | Large | CGAGAGATAC | 57,922 | 56,458 | 97.5 | 284 |
| **C** | PBS | Small | ACGAGTGCGT | 55,064 | 53,848 | 97.8 | 185 |
|  |  | Large | CGTGTCTCTA | 81,297 | 78,561 | 96.6 | 253 |
|  | PG | Small | TACACGTGAT | 35,637 | 23,042 | 64.7 | 99 |
|  |  | Large | TGTACTACTC | 65,352 | 56,126 | 85.9 | 256 |
| **D** | PBS | Small | CACGCTACGT | 32,728 | 31,364 | 95.8 | 105 |
|  |  | Large | AGCGTCGTCT | 100,764 | 96,496 | 95.8 | 339 |
|  | PG | Small | TACACACACT | 58,008 | 55,908 | 96.4 | 151 |
|  |  | Large | ACGCTCGACA | 63,095 | 56,970 | 90.3 | 267 |
| **E** | PBS | Small | CTCGCGTGTC | 44,910 | 44,270 | 98.6 | 156 |
|  |  | Large | CGAGAGATAC | 88,439 | 82,136 | 92.9 | 316 |
|  | PG | Small | ACATACGCGT | 48,374 | 45,649 | 94.4 | 137 |
|  |  | Large | AGTACGCTAT | 50,951 | 49,636 | 97.4 | 233 |
| **Total** |  |  |  | 1,171,101 | 1,107,491 | 94.6 |  |

^a^ Group A - group B and group C - group E were sequenced in separated FLX Titanium plates.
