# Supplementary material for: Alteration of Intestinal Microbiota in Mice Orally Administered with Salmon Cartilage Proteoglycan, a Prophylactic Agent
Source: PLoS One. 2013 Sep 9;8(9):e75008. doi: 10.1371/journal.pone.0075008 (PMC3767651; doi:10.1371/journal.pone.0075008)
Supplement: Table S2 — Distribution of the intestinal bacteria in the small intestine of PBS- and PG-administered mice at phylum-level. (DOCX) [file pone.0075008.s003.docx]

Table S2. Distribution of the intestinal bacteria in the **small** intestine of PBS- and PG-administered mice at phylum-level.

| **Phylum** | **Counts in small intestine**  **(% of total count)** | | | | | | | | | |
| --- | --- | --- | --- | --- | --- | --- | --- | --- | --- | --- |
|  | **Group A** | | **Group B** | | **Group C** | | **Group D** | | **Group E** | |
|  | **PBS** | **PG** | **PBS** | **PG** | **PBS** | **PG** | **PBS** | **PG** | **PBS** | **PG** |
| *Acidobacteria* | 0 | 1 | 0 | 0 | 0 | 0 | 0 | 0 | 0 | 0 |
|  | (0.000%) | (0.002%) | (0.000%) | (0.000%) | (0.000%) | (0.000%) | (0.000%) | (0.000%) | (0.000%) | (0.000%) |
| *Actinobacteria* | 702 | 2,520 | 351 | 180 | 120 | 225 | 209 | 430 | 135 | 612 |
|  | (1.361%) | (3.811%) | (0.667%) | (0.442%) | (0.223%) | (0.976%) | (0.666%) | (0.769%) | (0.305%) | (1.341%) |
| *Bacteroidetes* | 214 | 147 | 57 | 277 | 4,896 | 1,524 | 395 | 4,758 | 1,590 | 1,121 |
|  | (0.415%) | (0.222%) | (0.108%) | (0.680%) | (9.092%) | (6.614%) | (1.259%) | (8.510%) | (3.592%) | (2.456%) |
| *Chloroflexi* | 0 | 3 | 0 | 0 | 0 | 0 | 0 | 0 | 0 | 0 |
|  | (0.000%) | (0.005%) | (0.000%) | (0.000%) | (0.000%) | (0.000%) | (0.000%) | (0.000%) | (0.000%) | (0.000%) |
| *Deferribacteres* | 0 | 0 | 0 | 1 | 0 | 0 | 0 | 0 | 0 | 0 |
|  | (0.000%) | (0.000%) | (0.000%) | (0.002%) | (0.000%) | (0.000%) | (0.000%) | (0.000%) | (0.000%) | (0.000%) |
| *Firmicutes* | 17,217 | 42,231 | 49,987 | 31,095 | 45,027 | 16,046 | 27,525 | 40,875 | 39,643 | 39,337 |
|  | (33.386%) | (63.867%) | (94.935%) | (76.361%) | (83.619%) | (69.638%) | (87.760%) | (73.111%) | (89.548%) | (86.173%) |
| *Fusobacteria* | 0 | 2 | 0 | 0 | 0 | 0 | 0 | 0 | 0 | 0 |
|  | (0.000%) | (0.003%) | (0.000%) | (0.000%) | (0.000%) | (0.000%) | (0.000%) | (0.000) | (0.000%) | (0.000%) |
| *Proteobacteria* | 707 | 469 | 471 | 121 | 419 | 810 | 192 | 419 | 196 | 345 |
|  | (1.371%) | (0.709%) | (0.895%) | (0.297%) | (0.778%) | (3.515%) | (0.612%) | (0.749%) | (0.443%) | (0.756%) |
| *Spirochaetes* | 0 | 4 | 0 | 0 | 0 | 0 | 0 | 0 | 0 | 0 |
|  | (0.000%) | (0.006%) | (0.000%) | (0.000%) | (0.000%) | (0.000%) | (0.000%) | (0.000%) | (0.000%) | (0.000%) |
| *Tenericutes* | 1 | 0 | 0 | 0 | 1 | 2 | 0 | 1 | 0 | 0 |
|  | (0.002%) | (0.000%) | (0.000%) | (0.000%) | (0.002%) | (0.009%) | (0.000%) | (0.002%) | (0.000%) | (0.000%) |
| *Verrucomicrobia* | 23,964 | 15,324 | 181 | 504 | 0 | 0 | 0 | 0 | 0 | 0 |
|  | (46.470%) | (23.175%) | (0.344%) | (1.238%) | (0.000%) | (0.000%) | (0.000%) | (0.000%) | (0.000%) | (0.000%) |
| Unclassified | 8,764 | 5,422 | 1,607 | 8,543 | 3,385 | 4,435 | 3,043 | 9,425 | 2,706 | 4,234 |
|  | (16.995%) | (8.200%) | (3.052%) | (20.979%) | (6.286%) | (19.247%) | (9.702%) | (16.858%) | (6.112%) | (9.275%) |
| **Total** | **51,569** | **66,123** | **52,654** | **40,721** | **53,848** | **23,042** | **31,364** | **55,908** | **44,270** | **45,649** |
|  | **(100.00%)** | **(100.00%)** | **(100.00%)** | **(100.00%)** | **(100.00%)** | **(100.00%)** | **(100.00%)** | **(100.00%)** | **(100.00%)** | **(100.00%)** |
