# Supplementary material for: Alteration of Intestinal Microbiota in Mice Orally Administered with Salmon Cartilage Proteoglycan, a Prophylactic Agent
Source: PLoS One. 2013 Sep 9;8(9):e75008. doi: 10.1371/journal.pone.0075008 (PMC3767651; doi:10.1371/journal.pone.0075008)
Supplement: Table S3 — Distribution of the intestinal bacteria in the large intestine of PBS- and PG-administered mice at phylum-level. (DOCX) [file pone.0075008.s004.docx]

Table S3. Distribution of the intestinal bacteria in the **large** intestine of PBS- and PG-administered mice at phylum-level.

| **Phylum** | **Counts in large intestine**  **(% of total count)** | | | | | | | | | |
| --- | --- | --- | --- | --- | --- | --- | --- | --- | --- | --- |
|  | **Group A** | | **Group B** | | **Group C** | | **Group D** | | **Group E** | |
|  | **PBS** | **PG** | **PBS** | **PG** | **PBS** | **PG** | **PBS** | **PG** | **PBS** | **PG** |
| *Acidobacteria* | 0 | 0 | 0 | 0 | 0 | 0 | 0 | 0 | 0 | 0 |
|  | (0.000%) | (0.000%) | (0.000%) | (0.000%) | (0.000%) | (0.000%) | (0.000%) | (0.000%) | (0.000%) | (0.000%) |
| *Actinobacteria* | 672 | 587 | 217 | 375 | 733 | 130 | 985 | 615 | 832 | 1,502 |
|  | (1.211%) | (1.005%) | (0.417%) | (0.664%) | (0.933%) | (0.232%) | (1.021%) | (1.080%) | (1.013%) | (3.026%) |
| *Bacteroidetes* | 1,747 | 2,531 | 629 | 476 | 11,050 | 7,166 | 10,433 | 6,766 | 9,375 | 1,721 |
|  | (3.149%) | (4.334%) | (1.208%) | (0.843%) | (14.066%) | (12.768%) | (10.812%) | (11.876%) | (11.414%) | (3.467%) |
| *Chloroflexi* | 0 | 0 | 0 | 0 | 0 | 0 | 0 | 0 | 0 | 0 |
|  | (0.000%) | (0.000%) | (0.000%) | (0.000%) | (0.000%) | (0.000%) | (0.000%) | (0.000%) | (0.000%) | (0.000%) |
| *Deferribacteres* | 12 | 14 | 0 | 2 | 0 | 0 | 0 | 0 | 0 | 0 |
|  | (0.022%) | (0.024%) | (0.000%) | (0.004%) | (0.000%) | (0.000%) | (0.000%) | (0.000%) | (0.000%) | (0.000%) |
| *Firmicutes* | 32,030 | 22,416 | 42,884 | 52,275 | 54,416 | 41,572 | 72,825 | 45,464 | 64,570 | 44,161 |
|  | (57.733%) | (38.389%) | (82.330%) | (92.591%) | (69.266%) | (74.069%) | (75.469%) | (79.803%) | (78.614%) | (88.970%) |
| *Fusobacteria* | 0 | 0 | 0 | 0 | 0 | 0 | 0 | 0 | 0 | 0 |
|  | (0.000%) | (0.000%) | (0.000%) | (0.000%) | (0.000%) | (0.000%) | (0.000%) | (0.000%) | (0.000%) | (0.000%) |
| *Proteobacteria* | 612 | 330 | 192 | 229 | 1,261 | 686 | 465 | 585 | 497 | 222 |
|  | (1.103%) | (0.565%) | (0.369%) | (0.406%) | (1.605%) | (1.222%) | (0.482%) | (1.027%) | (0.605%) | (0.447%) |
| *Spirochaetes* | 1 | 10 | 0 | 0 | 0 | 0 | 0 | 0 | 0 | 0 |
|  | (0.002%) | (0.017%) | (0.000%) | (0.000%) | (0.000%) | (0.000%) | (0.000%) | (0.000%) | (0.000%) | (0.000%) |
| *Tenericutes* | 4 | 6 | 0 | 1 | 0 | 3 | 15 | 8 | 4 | 1 |
|  | (0.007%) | (0.010%) | (0.000%) | (0.002%) | (0.000%) | (0.005) | (0.016%) | (0.014%) | (0.005%) | (0.002%) |
| *Verrucomicrobia* | 9,478 | 23,571 | 3,449 | 612 | 0 | 0 | 0 | 0 | 0 | 0 |
|  | (17.084%) | (40.367%) | (6.621%) | (1.084%) | (0.000%) | (0.000%) | (0.000%) | (0.000%) | (0.000%) | (0.000%) |
| Unclassified | 10,924 | 8,927 | 4,717 | 2,488 | 11,101 | 6,569 | 11,773 | 3,532 | 6,858 | 2,029 |
|  | (19.690%) | (15.288%) | (9.056%) | (4.407%) | (14.130%) | (11.704%) | (12.201%) | (6.200%) | (8.350%) | (4.088%) |
| **Total** | **55,480** | **58,392** | **52,088** | **56,458** | **78,561** | **56,126** | **96,496** | **56,970** | **82,136** | **49,636** |
|  | **(100.00%)** | **(100.00%)** | **(100.00%)** | **(100.00%)** | **(100.00%)** | **(100.00%)** | **(100.00%)** | **(100.00%)** | **(100.00%)** | **(100.00%)** |
