# Supplementary material for: Alteration of Intestinal Microbiota in Mice Orally Administered with Salmon Cartilage Proteoglycan, a Prophylactic Agent
Source: PLoS One. 2013 Sep 9;8(9):e75008. doi: 10.1371/journal.pone.0075008 (PMC3767651; doi:10.1371/journal.pone.0075008)
Supplement: Table S4 — Distribution of mouse intestinal bacteria belonging to phylum Firmicutes in the small intestine of PBS- and PG-administered mice. (DOCX) [file pone.0075008.s005.docx]

Table S4. Distribution of mouse intestinal bacteria belonging to phylum *Firmicutes* in the **small** intestine of PBS- and PG-administered mice.

| **Class** | **Counts in small intestine**  **(% of total count)** | | | | | | | | | |
| --- | --- | --- | --- | --- | --- | --- | --- | --- | --- | --- |
|  | **Group A** | | **Group B** | | **Group C** | | **Group D** | | **Group E** | |
|  | **PBS** | **PG** | **PBS** | **PG** | **PBS** | **PG** | **PBS** | **PG** | **PBS** | **PG** |
| *Bacilli* | 640 | 1,725 | 1,124 | 1,715 | 27,115 | 5,519 | 2,934 | 24,766 | 9,542 | 10,589 |
|  | (1.241%) | (2.609%) | (2.135%) | (4.212%) | (50.355%) | (23.952%) | (9.355%) | (44.298%) | (21.554%) | (23.197%) |
| *Clostridia* | 16,487 | 39,696 | 3,067 | 11,824 | 11,562 | 5,094 | 22,968 | 15,482 | 28,419 | 27,770 |
|  | (31.971%) | (60.034%) | (5.825%) | (29.037%) | (21.472%) | (22.107%) | (73.230%) | (27.692%) | (64.195%) | (60.834%) |
| *Erysipelotrichi* | 42 | 288 | 45,769 | 17,384 | 6,347 | 5,432 | 1,623 | 626 | 1,679 | 977 |
|  | (0.081%) | (0.436%) | (86.924%) | (42.691%) | (11.787%) | (23.574%) | (5.175%) | (1.120%) | (3.793%) | (2.140%) |
| *Negativicutes* | 0 | 2 | 0 | 0 | 0 | 0 | 0 | 0 | 0 | 0 |
|  | (0.000%) | (0.003%) | (0.000%) | (0.000%) | (0.000%) | (0.000%) | (0.000%) | (0.000%) | (0.000%) | (0.000%) |
| Unclassified | 48 | 520 | 27 | 172 | 3 | 1 | 0 | 1 | 3 | 1 |
|  | (0.093%) | (0.786%) | (0.051%) | (0.422%) | (0.006%) | (0.004%) | (0.000%) | (0.002%) | (0.007%) | (0.002%) |
