# Supplementary material for: Alteration of Intestinal Microbiota in Mice Orally Administered with Salmon Cartilage Proteoglycan, a Prophylactic Agent
Source: PLoS One. 2013 Sep 9;8(9):e75008. doi: 10.1371/journal.pone.0075008 (PMC3767651; doi:10.1371/journal.pone.0075008)
Supplement: Table S5 — Distribution of mouse intestinal bacteria belonging to phylum Firmicutes in the large intestine of PBS- and PG-administered mice. (DOCX) [file pone.0075008.s006.docx]

Table S5. Distribution of mouse intestinal bacteria belonging to phylum *Firmicutes* in the **large** intestine of PBS- and PG-administered mice.

| **Class** | **Counts in large intestine**  **(% of total count)** | | | | | | | | | |
| --- | --- | --- | --- | --- | --- | --- | --- | --- | --- | --- |
|  | **Group A** | | **Group B** | | **Group C** | | **Group D** | | **Group E** | |
|  | **PBS** | **PG** | **PBS** | **PG** | **PBS** | **PG** | **PBS** | **PG** | **PBS** | **PG** |
| *Bacilli* | 656 | 2,077 | 1,065 | 2,380 | 5,073 | 1,342 | 10,992 | 22,418 | 11,807 | 13,274 |
|  | (1.182%) | (3.557%) | (2.045%) | (4.216%) | (6.457%) | (2.391%) | (11.391%) | (39.351%) | (14.375%) | (26.743%) |
| *Clostridia* | 30,835 | 19,682 | 7,739 | 7,498 | 48,895 | 39,305 | 60,246 | 22,332 | 51,094 | 30,390 |
|  | (55.579%) | (33.707%) | (14.858%) | (13.281%) | (62.238%) | (70.030%) | (62.434%) | (39.200%) | (62.207%) | (61.226%) |
| *Erysipelotrichi* | 108 | 59 | 34,018 | 42,339 | 364 | 780 | 1,441 | 658 | 1,614 | 494 |
|  | (0.195%) | (0.101%) | (65.309%) | (74.992%) | (0.463%) | (1.390%) | (1.493%) | (1.155%) | (1.965%) | (0.995%) |
| *Negativicutes* | 0 | 0 | 0 | 0 | 0 | 0 | 0 | 0 | 0 | 0 |
|  | (0.000%) | (0.000%) | (0.000%) | (0.000%) | (0.000%) | (0.000%) | (0.000%) | (0.000%) | (0.000%) | (0.000%) |
| Unclassified | 431 | 598 | 62 | 58 | 84 | 145 | 146 | 56 | 55 | 3 |
|  | (0.777%) | (1.024%) | (0.119%) | (0.103%) | (0.107%) | (0.258%) | (0.151%) | (0.098%) | (0.067%) | (0.006%) |
