# Supplementary material for: Alteration of Intestinal Microbiota in Mice Orally Administered with Salmon Cartilage Proteoglycan, a Prophylactic Agent
Source: PLoS One. 2013 Sep 9;8(9):e75008. doi: 10.1371/journal.pone.0075008 (PMC3767651; doi:10.1371/journal.pone.0075008)
Supplement: Table S6 — Bacterial phylotypes whose population level increased upon PG administration in the small intestine. (DOCX) [file pone.0075008.s007.docx]

Table S6. Bacterial phylotypes whose population level **increased** upon PG administration in the **small** intestine.

| **Phylum** | **Phylotype** | **Group A** | | **Group B** | | **Group C** | | **Group D** | | **Group E** | |
| --- | --- | --- | --- | --- | --- | --- | --- | --- | --- | --- | --- |
|  |  | **% Increase** | ***P* value^a^** | **% Increase** | ***P* value^a^** | **% Increase** | ***P* value^a^** | **% Increase** | ***P* value^a^** | **% Increase** | ***P* value^a^** |
| *Actinobacteria* | *Adlercreutzia equolifaciens* | 0.5105 | *P*<0.01 | -0.0460 | ND | 0.5067 | *P*<0.01 | 0.0015 | NS | 0.6549 | *P*<0.01 |
|  | *Enterorhabdus caecimuris* | 0.9868 | *P*<0.01 | -0.1168 | ND | 0.1966 | *P*<0.01 | 0.0800 | *P*<0.05 | 0.3574 | *P*<0.01 |
|  | *Enterorhabdus mucosicola* | 0.1285 | *P*<0.01 | 0.0159 | NS | 0.0099 | NS | 0.0202 | NS | 0.0348 | *P*<0.01 |
| *Bacteroidetes* | *Bacteroides acidifaciens* | 0.0076 | NS | 0.0055 | NS | 0.0006 | NS | 0.0089 | NS | -0.0113 | ND |
|  | *Bacteroides* sp. CJ44 | 0.0076 | NS | 0.0074 | NS | 0.0050 | NS | 0.0089 | NS | -0.0136 | ND |
|  | *Bacteroides* sp. SLC1-38 | 0.0030 | NS | 0.0311 | *P*<0.01 | 0.0260 | *P*<0.01 | 0.0072 | NS | -0.0091 | ND |
|  | *Alistipes putredinis* | 0.0197 | *P*<0.01 | 0.0213 | *P*<0.05 | 0.0304 | *P*<0.01 | 0.0125 | NS | -0.0090 | ND |
| *Firmicutes* | *Lactobacillus intestinalis* | 1.7233 | *P*<0.01 | 0.9481 | *P*<0.01 | -3.2336 | ND | 0.5232 | *P*<0.01 | 1.4798 | *P*<0.01 |
| Class *Bacilli* |  |  |  |  |  |  |  |  |  |  |  |
| *Firmicutes* | *Clostridium bolteae* | 0.1843 | *P*<0.01 | 0.5861 | *P*<0.01 | 0.0006 | NS | -0.0042 | ND | 0.0130 | NS |
| Class *Clostridia* | *Clostridium saccharolyticum* | 0.1121 | *P*<0.01 | 0.0502 | *P*<0.01 | 0.0267 | NS | 0.0022 | NS | -0.0025 | ND |
|  | *Clostridium* sp. ASF356 | 0.0011 | NS | 0.0797 | *P*<0.01 | 0.0044 | NS | 0.0004 | NS | -0.0045 | ND |
|  | *Clostridium* sp. Clone-17 | 0.1884 | *P*<0.01 | 0.2546 | *P*<0.01 | 0.0068 | NS | 0.0036 | NS | -0.0047 | ND |
|  | *Clostridium* sp. Clone-25 | 0.0259 | *P*<0.01 | 0.0589 | *P*<0.01 | -0.0019 | ND | 0.0036 | NS | 0.0042 | NS |
|  | *Clostridium* sp. Clone-44 | 0.0273 | *P*<0.05 | 0.0690 | *P*<0.01 | -0.0136 | ND | 0.0310 | *P*<0.05 | 0.0261 | *P*<0.05 |
|  | *Clostridium* sp. Clone-9 | 0.9941 | *P*<0.01 | 0.4948 | *P*<0.01 | 0.0930 | *P*<0.01 | 0.0179 | *P*<0.05 | -0.0068 | ND |
|  | *Clostridium* sp. Culture Jar-13 | 0.0002 | NS | 0.0281 | *P*<0.01 | 0.0403 | *P*<0.01 | 0.0093 | NS | 0.0020 | NS |
|  | *Clostridium* sp. Culture-41 | 0.6606 | *P*<0.01 | 1.4704 | *P*<0.01 | 0.0013 | NS | 0.0022 | NS | -0.0140 | ND |
|  | *Anaerovorax odorimutans* | 0.0385 | *P*<0.01 | 0.0336 | *P*<0.01 | -0.0006 | ND | 0.0294 | *P*<0.05 | 0.0172 | NS |
|  | *Eubacterium plexicaudatum* | 0.0091 | NS | 0.0074 | NS | -0.0241 | ND | 0.0036 | NS | 0.0022 | NS |
|  | *Ruminococcus gnavus* | -0.0015 | ND | 0.0025 | NS | 0.0025 | NS | 0.0022 | NS | 0.0022 | NS |
|  | Lachnospiraceae bacterium 607 | 0.0089 | NS | 0.0563 | *P*<0.01 | 0.0291 | *P*<0.01 | 0.0000 | NS | 0.0064 | NS |
|  | Lachnospiraceae bacterium A4 | 1.2964 | *P*<0.01 | 0.5819 | *P*<0.01 | 0.0914 | *P*<0.05 | 0.0107 | NS | 0.0394 | NS |
|  | Lachnospiraceae bacterium DJF_VP30 | 0.4851 | *P*<0.01 | 1.1441 | *P*<0.01 | 0.0025 | NS | 0.0194 | NS | -0.1356 | ND |
|  | Peptostreptococcaceae bacterium oral taxon 091 | -0.0019 | ND | 0.0074 | NS | 0.0006 | NS | 0.0004 | NS | 0.0022 | NS |
|  | *Ruminococcus* sp. CO28 | 0.1402 | *P*<0.01 | 0.2640 | *P*<0.01 | 0.1775 | *P*<0.01 | 0.1782 | *P*<0.01 | 0.2806 | *P*<0.01 |
|  | *Ruminococcus* sp. CO41 | 0.0238 | *P*<0.01 | 0.0606 | *P*<0.01 | -0.0093 | ND | 0.0018 | NS | 0.0065 | NS |
|  | Clostridiales bacterium oral taxon 085 | 0.0842 | *P*<0.01 | 0.3947 | *P*<0.01 | 0.0647 | *P*<0.05 | 0.0901 | *P*<0.01 | -0.0420 | ND |
| Unclassified | Gram-negative bacterium cL10-2b-4 | -9.3632 | ND | 15.5016 | *P*<0.01 | 12.7564 | *P*<0.01 | 6.8931 | *P*<0.01 | 3.1654 | *P*<0.01 |
|  | human intestinal firmicute CJ6 | 0.1220 | *P*<0.01 | 0.0516 | *P*<0.01 | -0.0037 | ND | 0.0133 | NS | 0.0197 | *P*<0.01 |
|  | TM7 phylum sp. oral taxon 351 | 0.0673 | *P*<0.01 | 0.3106 | *P*<0.01 | 0.2023 | *P*<0.01 | 0.2366 | *P*<0.01 | -0.0350 | ND |

^a^ Associations between bacterial phylotypes and PG administration were examined by Fisher exact test. *P* values less than 0.05 were used to indicate statistical difference of bacterial counts between PG-administered and control mice. NS: not significant difference. ND: not determined.
