# Supplementary material for: Alteration of Intestinal Microbiota in Mice Orally Administered with Salmon Cartilage Proteoglycan, a Prophylactic Agent
Source: PLoS One. 2013 Sep 9;8(9):e75008. doi: 10.1371/journal.pone.0075008 (PMC3767651; doi:10.1371/journal.pone.0075008)
Supplement: Table S7 — Bacterial phylotypes whose population level decreased upon PG administration in the small intestine. (DOCX) [file pone.0075008.s008.docx]

Table S7. Bacterial phylotypes whose population level **decreased** upon PG administration in the **small** intestine.

| **Phylum** | **Phylotype** | **Group A** | | **Group B** | | **Group C** | | **Group D** | | **Group E** | |
| --- | --- | --- | --- | --- | --- | --- | --- | --- | --- | --- | --- |
|  |  | **% Increase** | ***P* value^a^** | **% Increase** | ***P* value^a^** | **% Increase** | ***P* value^a^** | **% Increase** | ***P* value^a^** | **% Increase** | ***P* value^a^** |
| *Actinobacteria* | *Gordonibacter pamelaeae* | 0.3654 | *P*<0.01 | 0.0086 | NS | -0.0372 | ND | 0.0205 | *P*<0.01 | 0.0228 | *P*<0.05 |
| *Firmicutes* | *Lactobacillus reuteri* | 0.0582 | NS | 0.1159 | *P*<0.01 | 2.3048 | *P*<0.01 | -2.3052 | ND | 0.2356 | *P*<0.05 |
| Class *Bacilli* | *Lactobacillus* sp. ID9203 | 0.0213 | *P*<0.01 | 0.0114 | NS | 0.1820 | *P*<0.01 | 0.0023 | NS | 0.0025 | NS |
|  | *Lactococcus lactis* | 0.0019 | NS | 0.0095 | NS | 0.0427 | *P*<0.01 | -0.0036 | ND | 0.0023 | NS |
| *Firmicutes* | *Clostridium* sp. Culture-54 | 0.0021 | NS | -0.1482 | ND | 0.0285 | NS | 0.0080 | NS | 0.0029 | NS |
| Class *Clostridia* | *Clostridium* sp. ID4 | 6.5279 | *P*<0.01 | 0.0142 | NS | 4.3533 | *P*<0.01 | 44.7604 | *P*<0.01 | 1.6573 | *P*<0.01 |

^a^ Associations between bacterial phylotypes and PG administration were examined by Fisher exact test. *P* values less than 0.05 were used to indicate statistical difference of bacterial counts between PG-administered and control mice. NS: not significant difference. ND: not determined.
