# Supplementary material for: Alteration of Intestinal Microbiota in Mice Orally Administered with Salmon Cartilage Proteoglycan, a Prophylactic Agent
Source: PLoS One. 2013 Sep 9;8(9):e75008. doi: 10.1371/journal.pone.0075008 (PMC3767651; doi:10.1371/journal.pone.0075008)
Supplement: Table S8 — Bacterial phylotypes whose population level increased upon PG administration in the large intestine. (DOCX) [file pone.0075008.s009.docx]

Table S8. Bacterial phylotypes whose population level **increased** upon PG administration in the **large** intestine.

| **Phylum** | **Phylotype** | **Group A** | | **Group B** | | **Group C** | | **Group D** | | **Group E** | |
| --- | --- | --- | --- | --- | --- | --- | --- | --- | --- | --- | --- |
|  |  | **% Increase** | ***P* value^a^** | **% Increase** | ***P* value^a^** | **% Increase** | ***P* value^a^** | **% Increase** | ***P* value^a^** | **% Increase** | ***P* value^a^** |
| *Bacteroidetes* | *Odoribacter splanchnicus* | 0.2260 | *P*<0.01 | 0.0049 | NS | 0.1975 | *P*<0.01 | 0.0511 | *P*<0.01 | -0.0183 | ND |
|  | *Parabacteroides distasonis* | 0.0882 | *P*<0.01 | 0.0153 | NS | 0.2011 | *P*<0.01 | 0.0557 | *P*<0.01 | -0.1572 | ND |
|  | *Prevotella* sp. canine oral taxon 298 | 0.0032 | NS | 0.0153 | NS | 0.7040 | *P*<0.01 | 0.3206 | *P*<0.01 | -1.1303 | ND |
| *Firmicutes* | *Lactobacillus intestinalis* | 1.8518 | *P*<0.01 | 1.9067 | *P*<0.01 | -0.8585 | ND | 0.2068 | *P*<0.01 | 2.2112 | *P*<0.01 |
| Class *Bacilli* | *Lactobacillus johnsonii* | 0.1404 | *P*<0.01 | 0.2216 | *P*<0.01 | -0.2488 | ND | 16.4979 | *P*<0.01 | 8.5695 | *P*<0.01 |
|  | *Lactobacillus reuteri* | 1.0813 | *P*<0.01 | 0.1460 | *P*<0.01 | -0.3305 | ND | 1.2783 | *P*<0.01 | 1.1188 | *P*<0.01 |
| *Firmicutes* | *Clostridium bolteae* | -0.4745 | ND | 0.0401 | *P*<0.01 | 0.0547 | *P*<0.05 | 0.0699 | *P*<0.01 | 0.0636 | *P*<0.01 |
| Class *Clostridia* | *Clostridium fusiformis* | 0.0395 | NS | 0.2332 | *P*<0.01 | 1.0613 | *P*<0.01 | -0.3193 | ND | 0.1349 | NS |
|  | *Clostridium* sp. strain Z6 | 0.1776 | *P*<0.01 | 0.0143 | NS | 0.0043 | NS | 0.0062 | NS | -0.0565 | ND |
|  | *Blautia glucerasea* | 0.0120 | *P*<0.05 | 0.0068 | NS | 0.0005 | NS | 0.0018 | NS | -0.0016 | ND |
|  | *Roseburia intestinalis* | 0.0034 | NS | 0.0624 | *P*<0.01 | 0.6999 | *P*<0.01 | -0.0119 | ND | 0.1238 | *P*<0.01 |
|  | *Desulfotomaculum* sp. CYP1 | 0.1386 | *P*<0.01 | 0.0087 | NS | -0.0051 | ND | 0.0483 | *P*<0.01 | 0.0135 | NS |
|  | *Ruminococcus* sp. M10 | 0.0051 | NS | 0.0596 | *P*<0.01 | -0.0038 | ND | 0.4534 | *P*<0.01 | 0.0031 | NS |
|  | *Eubacterium tortuosum* | 0.0017 | NS | 0.0071 | NS | 0.0071 | NS | -0.0021 | ND | 0.0008 | NS |
| Unclassified | anaerobic bacterium U3/P-1 | 0.0016 | NS | 0.0016 | NS | 0.0000 | NS | 0.0007 | NS | 0.0036 | NS |
|  | rumen bacterium NK4C38 | 0.0051 | NS | 0.0034 | NS | 0.0010 | NS | 0.0085 | NS | -0.0024 | ND |

^a^ Associations between bacterial phylotypes and PG administration were examined by Fisher exact test. *P* values less than 0.05 were used to indicate statistical difference of bacterial counts between PG-administered and control mice. NS: not significant difference. ND: not determined.
