# Supplementary material for: Alteration of Intestinal Microbiota in Mice Orally Administered with Salmon Cartilage Proteoglycan, a Prophylactic Agent
Source: PLoS One. 2013 Sep 9;8(9):e75008. doi: 10.1371/journal.pone.0075008 (PMC3767651; doi:10.1371/journal.pone.0075008)
Supplement: Table S9 — Bacterial phylotypes whose population level decreased upon PG administration in the large intestine. (DOCX) [file pone.0075008.s010.docx]

Table S9. Bacterial phylotypes whose population level **decreased** upon PG administration in the **large** intestine.

| **Phylum** | **Phylotype** | **Group A** | | **Group B** | | **Group C** | | **Group D** | | **Group E** | |
| --- | --- | --- | --- | --- | --- | --- | --- | --- | --- | --- | --- |
|  |  | **% Increase** | ***P* value^a^** | **% Increase** | ***P* value^a^** | **% Increase** | ***P* value^a^** | **% Increase** | ***P* value^a^** | **% Increase** | ***P* value^a^** |
| *Bacteroidetes* | *Barnesiella* sp. NSB1 | 0.4027 | *P*<0.01 | 0.0180 | NS | 8.1716 | *P*<0.01 | 2.1846 | *P*<0.01 | 2.8894 | *P*<0.01 |
| *Firmicutes* | *Clostridium glycyrrhizinilyticum* | 0.0055 | NS | 0.0058 | NS | -0.0023 | ND | 0.0037 | NS | 0.0016 | NS |
| Class *Clostridia* | *Clostridium hathewayi* | 0.1975 | *P*<0.01 | -0.2094 | ND | 0.1482 | *P*<0.01 | 0.0585 | *P*<0.01 | 0.0700 | *P*<0.05 |
|  | *Clostridium* sp. A9 | 3.9315 | *P*<0.01 | 0.1768 | *P*<0.01 | 0.5680 | *P*<0.01 | 0.0954 | *P*<0.01 | -0.0635 | ND |
|  | *Clostridium* sp. ASF502 | 0.9977 | *P*<0.01 | -0.3950 | ND | 0.1837 | *P*<0.01 | 0.4374 | *P*<0.01 | 0.0862 | NS |
|  | *Clostridium* sp. Clone-16 | 5.0237 | *P*<0.01 | 1.5652 | *P*<0.01 | 0.0555 | *P*<0.01 | -0.7290 | ND | 0.0049 | NS |
|  | *Clostridium* sp. Clone-17 | 0.8990 | *P*<0.01 | -0.1466 | ND | 0.0451 | NS | 0.0790 | *P*<0.01 | 0.0659 | *P*<0.01 |
|  | *Clostridium* sp. Clone-25 | 0.0039 | NS | 0.0024 | NS | 0.0229 | NS | 0.0263 | *P*<0.01 | 0.0715 | *P*<0.01 |
|  | *Clostridium* sp. Clone-27 | 0.0819 | *P*<0.01 | 0.4378 | *P*<0.01 | 0.6657 | *P*<0.01 | 6.1485 | *P*<0.01 | 0.0230 | NS |
|  | *Clostridium* sp. Clone-33 | 0.0000 | ND | 0.0136 | *P*<0.05 | 0.0369 | *P*<0.01 | 0.0974 | *P*<0.01 | 0.0536 | *P*<0.01 |
|  | *Clostridium* sp. Clone-44 | 0.4987 | *P*<0.01 | 0.0740 | *P*<0.01 | 0.0292 | NS | 0.1025 | *P*<0.05 | -0.1104 | ND |
|  | *Clostridium* sp. Clone-45 | -0.0017 | ND | 0.0038 | NS | 0.0028 | NS | 0.0073 | NS | 0.0085 | NS |
|  | *Clostridium* sp. Clone-47 | 0.0149 | NS | 0.0223 | NS | -0.7940 | ND | 0.0905 | *P*<0.01 | 0.3953 | *P*<0.01 |
|  | *Clostridium* sp. Clone-7 | 0.0349 | *P*<0.01 | 0.0061 | NS | 0.0229 | *P*<0.01 | 0.0587 | *P*<0.01 | 0.0568 | *P*<0.01 |
|  | *Clostridium* sp. Culture Jar-15 | 0.0168 | NS | 0.0087 | NS | -0.0455 | ND | 0.0320 | *P*<0.05 | 0.0105 | NS |
|  | *Clostridium* sp. Culture Jar-17 | 0.0055 | NS | -0.0013 | ND | 0.0064 | NS | 0.0249 | *P*<0.01 | 0.0130 | NS |
|  | *Clostridium* sp. Culture-1 | 0.1438 | *P*<0.01 | 0.0956 | *P*<0.01 | -0.0341 | ND | 0.0867 | *P*<0.01 | 0.0906 | *P*<0.01 |
|  | *Clostridium* sp. Culture-27 | 0.0346 | NS | 0.0192 | *P*<0.01 | -0.0621 | ND | 0.3233 | *P*<0.01 | 0.0820 | *P*<0.01 |
|  | *Clostridium xylanolyticum* | 0.9237 | *P*<0.01 | 0.0407 | *P*<0.05 | 0.0533 | NS | 0.2812 | *P*<0.01 | -0.2068 | ND |
|  | Clostridiaceae bacterium WN011 | -0.0034 | ND | 0.0058 | NS | 0.0013 | NS | 0.0021 | NS | 0.0730 | *P*<0.01 |
|  | *Eubacterium coprostanoligenes* | 0.2865 | *P*<0.01 | 1.4046 | *P*<0.01 | 0.2890 | *P*<0.01 | -0.7376 | ND | 0.2991 | *P*<0.01 |
|  | *Eubacterium plexicaudatum* | -0.0924 | ND | 0.0115 | *P*<0.05 | 1.1043 | *P*<0.01 | 0.0483 | NS | 0.1301 | *P*<0.01 |
|  | *Ruminococcus gnavus* | 0.0280 | *P*<0.01 | 0.0505 | *P*<0.01 | -0.0522 | ND | 0.3308 | *P*<0.01 | 0.0725 | *P*<0.01 |
|  | *Roseburia faecis* | 0.0077 | NS | -0.0177 | ND | 0.0812 | *P*<0.01 | 0.0496 | *P*<0.01 | 0.0451 | *P*<0.01 |
|  | Lachnospiraceae bacterium 3-1 | 0.0382 | *P*<0.01 | 0.0059 | NS | -0.0071 | ND | 0.0010 | NS | 0.0061 | NS |

Table S9. (Continue)

| **Phylum** | **Phylotype** | **Group A** | | **Group B** | | **Group C** | | **Group D** | | **Group E** | |
| --- | --- | --- | --- | --- | --- | --- | --- | --- | --- | --- | --- |
|  |  | **% Increase** | ***P* value^a^** | **% Increase** | ***P* value^a^** | **% Increase** | ***P* value^a^** | **% Increase** | ***P* value^a^** | **% Increase** | ***P* value^a^** |
| *Firmicutes* | Lachnospiraceae bacterium 6-1 | 0.0061 | NS | 0.0157 | *P*<0.05 | 0.0741 | *P*<0.01 | 0.0216 | NS | -0.0153 | ND |
| Class *Clostridia* | Lachnospiraceae bacterium A2 | 0.0018 | NS | 0.0044 | NS | -0.2907 | ND | 0.0065 | NS | 0.0090 | NS |
|  | Lachnospiraceae bacterium DJF_VP30 | 1.0456 | *P*<0.01 | 0.3284 | *P*<0.01 | -0.2884 | ND | 0.6343 | *P*<0.01 | 1.7169 | *P*<0.01 |
|  | *Oscillibacter* sp. G2 | 0.3307 | *P*<0.01 | -0.0587 | ND | 0.0474 | *P*<0.05 | 0.1391 | *P*<0.01 | 0.1729 | *P*<0.01 |
|  | *Oscillibacter valericigenes* | 0.5170 | *P*<0.01 | 0.0297 | NS | 0.0344 | *P*<0.01 | 0.2595 | *P*<0.01 | 0.0552 | *P*<0.01 |
|  | Peptostreptococcaceae bacterium canine oral taxon 125 | 0.0163 | *P*<0.01 | 0.0038 | NS | 0.0008 | NS | 0.0021 | NS | 0.2013 | *P*<0.01 |
|  | Peptostreptococcaceae bacterium canine oral taxon 221 | -0.0017 | ND | 0.0001 | NS | 0.0102 | *P*<0.05 | 0.0014 | NS | 0.0414 | *P*<0.01 |
|  | *Ruminococcus flavefaciens* | 0.0315 | NS | 0.0600 | *P*<0.01 | -0.0804 | ND | 0.0237 | NS | 0.1849 | *P*<0.01 |
|  | *Ruminococcus* sp. CJ63 | -0.0202 | ND | 0.0366 | *P*<0.01 | 0.0041 | NS | 0.0062 | NS | 0.0024 | NS |
|  | Clostridiales bacterium 21-4c | 0.0857 | *P*<0.01 | -0.0105 | ND | 0.0575 | *P*<0.01 | 0.0050 | NS | 0.1126 | *P*<0.01 |
|  | Clostridiales bacterium 24-4c | 0.0388 | *P*<0.01 | 0.0024 | NS | 0.0043 | NS | 0.0016 | NS | 0.0073 | NS |
|  | Clostridiales bacterium 37-2a | 0.0000 | ND | 0.0173 | *P*<0.01 | 0.0163 | *P*<0.05 | 0.0155 | *P*<0.05 | 0.0025 | NS |
|  | *Flavonifractor plautii* | 0.0219 | *P*<0.01 | 0.0117 | NS | 0.0000 | ND | 0.0021 | NS | 0.0037 | NS |
|  | bacterium ASF500 | 0.0519 | NS | 0.0231 | NS | -0.0624 | ND | 0.0024 | NS | 0.0171 | *P*<0.05 |
| *Proteobacteria* | *Parasutterella excrementihominis* | 0.1167 | *P*<0.01 | 0.1249 | *P*<0.01 | 0.5211 | *P*<0.01 | -0.5462 | ND | 0.1136 | *P*<0.01 |
| Unclassified | Gram-negative bacterium cL10-2b-4 | 4.7331 | *P*<0.01 | 4.0530 | *P*<0.01 | 4.4887 | *P*<0.01 | 6.0655 | *P*<0.01 | 4.1118 | *P*<0.01 |
|  | human intestinal firmicute CJ7 | 0.1602 | *P*<0.01 | -0.0035 | ND | 0.0003 | NS | 0.0117 | NS | 0.0113 | NS |

^a^ Associations between bacterial phylotypes and PG administration were examined by Fisher exact test. *P* values less than 0.05 were used to indicate statistical difference of bacterial counts between PG-administered and control mice. NS: not significant difference. ND: not determined.
